# Negative pressure wound therapy promotes healing and reduced pain in patients with acute suppurative mastitis

**DOI:** 10.1186/s12905-022-01785-z

**Published:** 2022-06-18

**Authors:** Lin Qiu, Feng Wang, Qin Xu, Zhenlv Lin, Bo Lin, Meng Huang, Qiaoyi Wu

**Affiliations:** 1 Department of Trauma Center and Emergency Surgery, The First Affiliated Hospital of Fujian Medical University, 20 Chazhong Road, Fujian 350004 Fuzhou, People’s Republic of China; 2 Department of Outpatient Care, Fujian Hospital of People’s Armed Police, Fuzhou, 350025 People’s Republic of China; 3 Department of Gynecology, Fujian Cancer Hospital and Fujian Medical University Cancer Hospital, Fuzhou, 350014 People’s Republic of China; 4 Fujian Center for Disease Control and Prevention, Fuzhou, 350001 People’s Republic of China

**Keywords:** Acute suppurative mastitis, Vacuum sealing drainage, Treatment

## Abstract

**Background:**

The purpose of this study was to compare the outcomes of vacuum sealing drainage (VSD) and conventional incision and drainage (I&D) for treating acute suppurative mastitis.

**Methods:**

Hospital medical records were searched for patients 20–50 years of age who were diagnosed with acute suppurative mastitis from January 2014 to December 2018, and treated with traditional I&D or VSD. Patients were divided into those treated with VSD and I&D, and outcomes including pain, healing time, length of hospital stay, and length of antibiotic course were compared between the groups. Pain was evaluated with a numeric rating scale from 0 (no pain) to 10 (most severe pain). Subgroup analysis of lactating women was also performed.

**Results:**

There were 110 women who received traditional I&D, and 105 women that received VSD included. The 2 groups were similar with respect to age (31.1 ± 4.8 vs. 29.9 ± 4.4, *p* = 0.058), and disease characteristics. The median pain score of women who received VSD (5 [IQR 5–6]) was significantly less than that of women who received I&D (8 [IQR 7–8]) (*p* < 0.001). The time for healing was significantly less in women who received VSD (40 days [IQR 30–45 days]) compared to I&D (60 days [IQR 45–70 days]) (*p* < 0.001). The length of hospital say and the length of antibiotic treatment were similar between the 2 groups. Results were similar for lactating women.

**Conclusions:**

VSD is effective for treating acute suppurative mastitis with reduced pain and shortening healing time.

## Background

Breast infections in women usually occur between 18 and 50 years of age [1]. A breast abscess, normally formed as a consequence of mastitis, affects 0.4–11% of breastfeeding women [2]. Breast abscesses are usually treated by surgical drainage. However, the conventional incision and drainage (I&D) is typically associated with excessive exudation, the wound requires frequent dressing changes that can be painful, and thus the workload for hospital staff is increased.

Vacuum sealing drainage (VSD) is a novel and effective technique that has been used to treat and promote healing of various types of wounds [3–5]. VSD has been shown to be successful in the treatment of osteofascial compartment syndrome and dermatoplasty of large areas of cutaneous defects [6, 7]. These wounds are characterized by an excessive amount exudation and necrosis, and with traditional wound care (i.e., dressing changes) these wounds often take a long time to heal. Alternatively, VSD uses negative pressure suction to alleviate wound exudation and stimulate the growth of granulation tissue.

Thus, the purpose of this study was to compare the outcomes of VSD and conventional I&D for the treatment of acute suppurative mastitis. Outcomes were assessed in both lactating and non-lactating women. We hypothesized that VSD would result in lower pain levels and faster healing rates compared to conventional therapy.

## Methods

### Patient information

The study was approved by the Ethics Committee of the First Affiliated Hospital of Fujian Medical University ([2020]045).

We included patients who are acute suppurative mastitis without other complications after surgical treatment. The diagnostic criteria for acute suppurative mastitis were high fever, chills, breast redness and tenderness, and ultrasound confirmation of a breast abscess. Other complications have two layers of meaning, one of which is co-morbid medical conditions such as diabetes mellitus, severe heart, kidney, liver, or blood diseases, malignancies or tumors and the other of which is co-infectious diseases such as respiratory infections, gastroenteritis, cholecystitis, encephalitis, other infectious diseases. The surgical treatment is defined as conventional incision and drainage (I&D) or negative pressure wound therapy (VSD).

### Treatments

All patients were treated with antibiotics, combined with conventional I&D or negative pressure wound therapy (i.e., VSD). Acute suppurate mastitis is usually associated with a gram positive bacterial infection. The most common pathogens in acute, subacute, and granulomatous mastitis are *Staphylococcus aureus*, *Staphylococcus epidermidis*, and *Corynebacteria species*, respectively [8]. Thus, patients were empirically treated with a third generation cephalosporin such as cefotaxime [8]. A quinolones was used for patients with a known reaction to cephalosporins. Wound swabs were generally sent for bacterial culture and sensitivity testing, and the choice of antibiotics was then adjusted according to the culture and sensitivity results and the patient’s condition.

Patients chose which treatment they wanted after a discussion with their physician. In some cases, a patient’s decision was based on economic concerns as traditional I&D is less costly than VSD.

For analysis, patients were grouped according to those that received traditional I&D and those that received VSD. Briefly, with traditional I&D a small incision required full exposure to drain the wound thoroughly is made where the abscess is most obvious (usually after injection of a local anesthetic). The abscess contents are expressed, and the cavity is repeatedly irrigated with 0.9% saline and hydrogen peroxide until it is basically clean and no pus is visible.The wound is packed with gauze soaked in a chlorinated lime and boric acid solution and then covered with dry gauze. The wound packing and dressing is changed daily until the wound fully closed [9].

With VSD, a much smaller incision than that with traditional I&D is made where the abscess is most obvious, which is large enough for the polyethylene alcohol hydration foam to pass through (after local anesthesia is administered). The wound is debrided, and irrigated with 0.9% saline and hydrogen peroxide until it is basically clean and no pus is visible. It is then completely covered with polyethylene alcohol hydration foam (Guangdong Meijie Weitong Biotechnology Co., LTD, China). A semi-permeable membrane is then pasted onto the surface of the foam and the surrounding normal skin for sealing and fixing. After operation, they are connected to central negative pressure system, and continuous negative pressure at 150 mmHg is connected and the wound is irrigated with 0.9% saline through a side tube. If the foam expands, it suggests that negative pressure aspiration is not occurring, and that there are leaks that need to be identified and repaired [10]. After 3 days, the membrane and foam are removed and the wound is inspected. Repeat debridement is performed if necessary. The foam and membrane are replaced, and VSD is begun again. The process is repeated until the wound develops healthy granulation tissue.

### Outcome evaluation

Data extracted from the medical records included patient age and medical history, disease history, the number and type of abscesses, whether or not the patient was lactating, bacterial culture results, pathological results including a diagnosis of plasma cell mastitis or not plasma cell mastitis if tissue was sent for examination, the type of treatment, length of hospital stay, and length of time receiving antibiotics. As patients were discharged before wounds were completely healed, pain level at the time of wound dressing changes and the time for wound to completely heal were obtained by telephone interview.

Pain level was evaluated at the time of wound dressing changes or VSD change using a numerical rating scale (NRS) ranging from 0 to 10. A score of 0–3 was considered mild pain, 4–7 was moderate pain, and 8–10 was severe pain. Healing time was defined as the time from abscess drainage to complete wound closure. If patients were discharged before complete wound closure, information was obtained by telephone follow-up. The length of the hospital stay was defined as the time from admission until hospital discharge. Antibiotic treatment course was defined as the time antibiotics were begun (which was usually at admission) to the time they were discontinued. Antibiotics were discontinued when the breast mass/pain had resolved and body temperature and white blood cell (WBC) count had returned to normal.

The subgroups of patients who were lactating were also compared. That is, the outcomes of lactating women who were treated with I&D were compared with lactating women who were treated with VSD.

### Included criteria

The patients those met the following inclusion criteria were adopted for our study: (1) Acute suppurative mastitis with ultrasound confirmation of a breast abscess at least; (2) Aging from 20 to 50; (3) Patients who visited our hospital from January 2014 to December 2018; (4) Treated with traditional I&D or VSD;5. Willing to cooperate to participate in the study and accept follow-up.

### Exclusion criteria

This paper excluded the relevant patients based on the following: 1. Irrelevant patients who are not diagnosed acute suppurative mastitis; 2. Patients diagnosed acute supprative mastitis without abscess cavity formation; 3. Patients with co-morbid medical conditions such as diabetes, sever heart disease, kidney disease, liver disease, blood disease, or tumors; 4. Patients with respitatory infections, gastroenteritis, cholecystitis, encephalitis, or other infectious diseases; 5. Treated conservatively; 6. Operated upon by other surgical teams; 7. Unwilling to cooperate with follow-up; 8.Older than 50 or younger than 20; 9. The relevant time cannot be recalled.

### Statistical analysis

The Kolmogorov-Smirnov method was used to detect the normality of the data. Categorical data were expressed and number and percentage, and comparisons between 2 groups were performed with Fisher’s exact test when the number in the table was < 40, and the chi-square (χ^2^) test when number was > 40. Continuous data were expressed as mean ± standard deviation, and normally distributed data were compared with the t test. Non-normally distributed data were presented as median and interquartile range (IQR), and examined with the Wilcoxon rank sum test. Statistical analyses were performed with SPSS version 19.0 software (SPSS Inc., USA). Values of *p* < 0.05 were considered to indicate statistical significance.

## Results

**Fig. 1:**
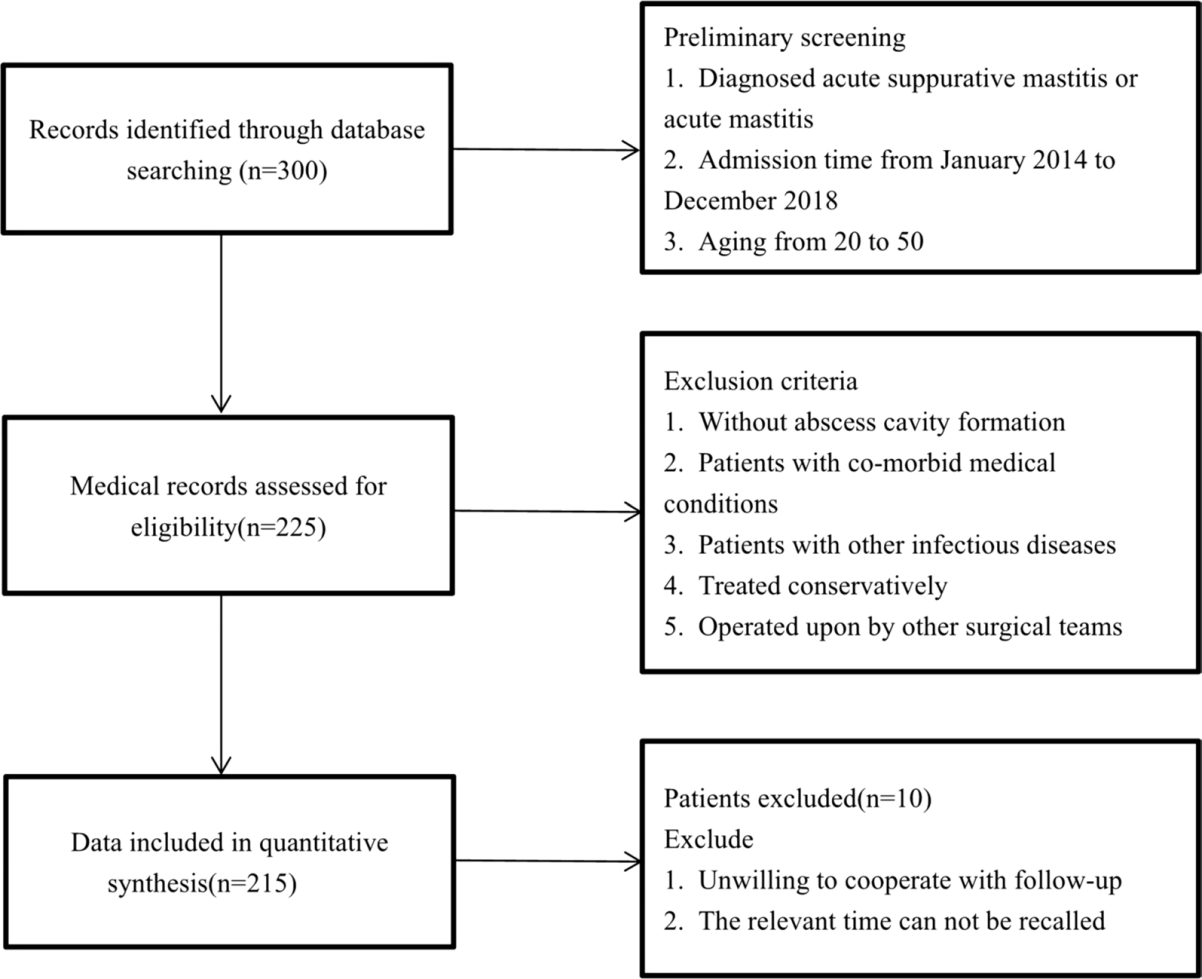
Process of patients selection

A total of 300 patients were retrieved from the electronic data-bases who are diagnosed acute mastitis, acute suppurative mastitis or breast abscess from January 2014 to December 2018 in our hospital. We excluded 75 on the exclusion criteria, and the remaining 225 patients were retrieved for more detailed evaluation. Based on follow-up, 215 patients fulfilled the criteria for eligibility and were included in the review (Fig. 1).

There were 110 women who received traditional I&D, and 105 women that received VSD and irrigation with physiological saline. As shown in Table 1, the 2 groups were similar with respect to age (31.1 ± 4.8 vs. 29.9 ± 4.4, p = 0.058), as well as the numbers of patients with single vs. multiple abscesses and the numbers of patients with plasma cell mastitis vs. not plasma cell mastitis (both, *p* > 0.05).

**Table 1:** Characteristics of the incision and drainage group and the vacuum sealing drainage group

	Incision and drainage (n = 110)	Vacuum sealing drainage (n = 105)	*p*
Age, years	31.1 ± 4.8	29.9 ± 4.4	0.058
Single abscess	96	91	0.895
Multiple abscess	14	14	
Not plasma cell mastitis	101	97	0.879
Plasma cell mastitis	9	8	

Age presented as mean ± standard deviation; other data as count

Clinical outcomes of the 2 groups are summarized in Table 2. The median pain score of women who received VSD (5 [IQR 5–6]) was significantly less than that of women who received I&D (8 [IQR 7–8]) (*p* < 0.001). In addition, the time for healing was significantly less in women who received VSD (40 days [IQR 30–45 days]) than in those that received I&D (60 days [IQR 45–70 days]) (*p* < 0.001). The length of hospital stay and the length of antibiotic treatment were similar between the 2 groups (Table 2).

**Table 2:** Clinical outcomes of the incision and drainage group and the vacuum sealing drainage group

	Incision and drainage (n = 110)	Vacuum sealing drainage (n = 105)	*p*
Pain score	8 (7–8)	5 (5–6)	< 0.001
Hospital stay, days	9.9 ± 2.5	10.1 ± 2.4	0.483
Healing time, days	60 (45–70)	40 (30–45)	< 0.001
Antibiotic treatment course, days	10.1 ± 2.2	10.2 ± 2.1	0.780

Data reported as mean ± standard deviation, or median (interquartile range)

**Fig. 2:**
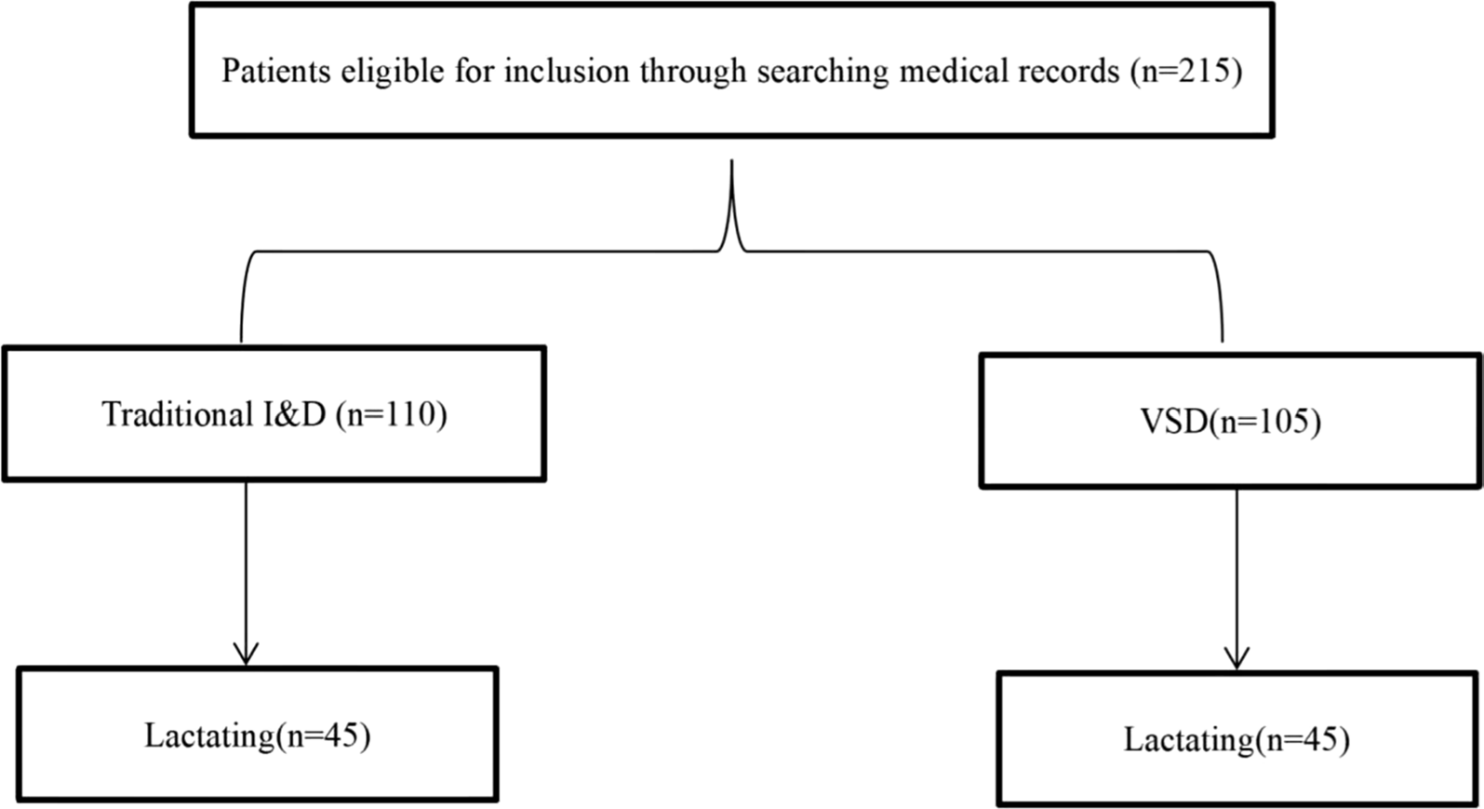
The group distribution of included patients

Results of the subgroup analysis of lactating women are shown in Table 3. There were 45 women who received I&D that were lactating and 45 women that received VSD that were lactating (Fig. 2). The median pain score of lactating women who received VSD (5 [IQR 5–6]) was significantly less than that of lactating women who received I&D (8 [IQR 7–8]) (*p* < 0.001). In addition, the time for healing was significantly less in lactating women who received VSD (40 days [IQR 30–50 days]) than in lactating women who received I&D (60 days [IQR 45–70 days]) (*p* < 0.001). The length of hospital say and the length of antibiotic treatment were similar between the 2 groups (Table 3).

**Table 3:** Clinical outcomes of lactating women who received incision and drainage or received vacuum sealing drainage

	Incision and drainage, lactating (n = 45)	Vacuum sealing drainage, lactating (n = 45)	*p*
Pain score	8 (7–8)	5 (5–6)	< 0.001
Hospital stay, days	10.0 ± 2.1	9.9 ± 2.3	0.774
Healing time, days	60 (45–70)	40 (30–50)	< 0.001
Antibiotic treatment course, days	10.3 ± 2.0	9.8 ± 2.1	0.311

Data reported as mean ± standard deviation, or median (interquartile range)

**Fig. 3:**
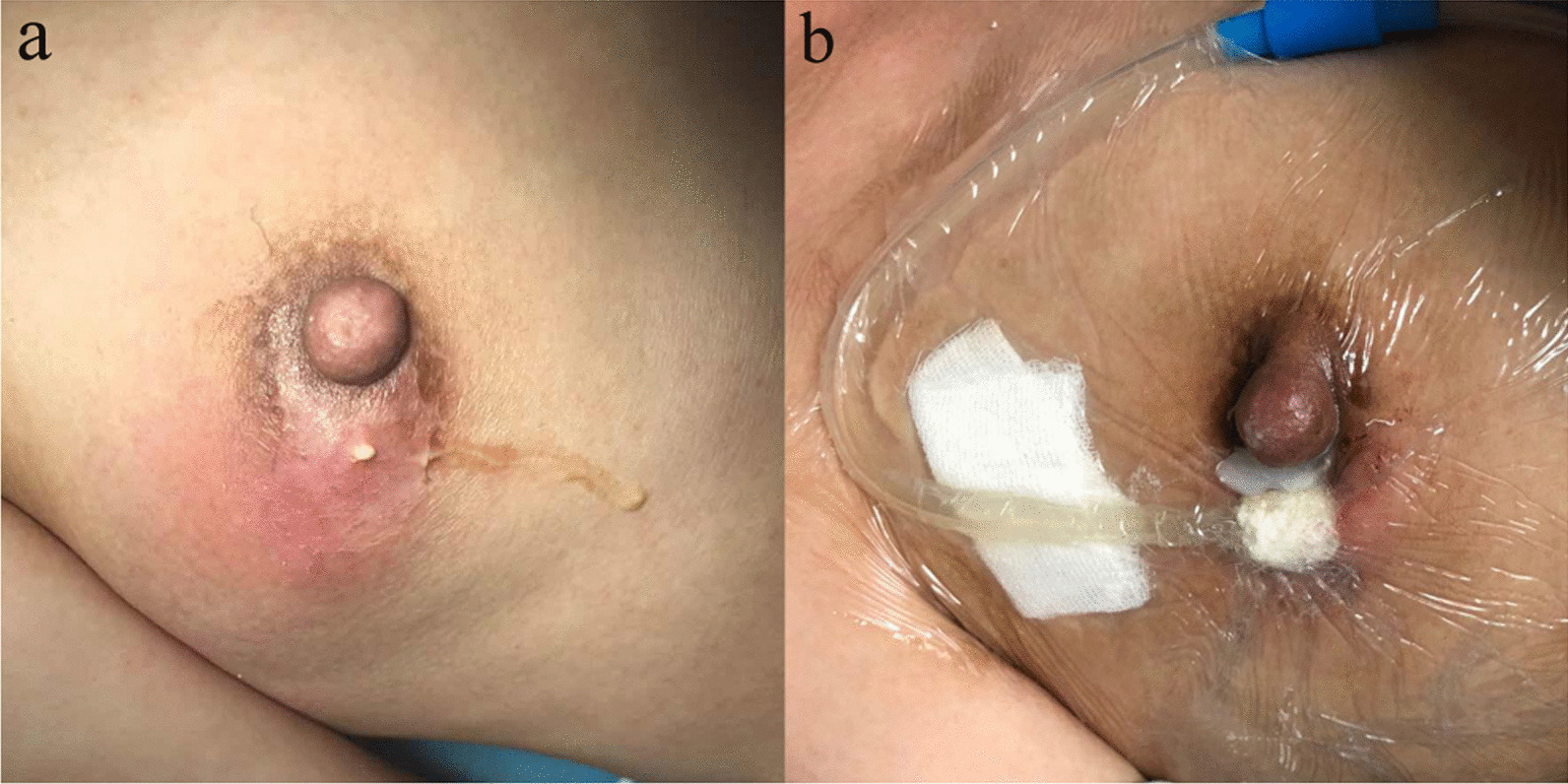
**A** Preoperative breast abscess. **B** After 3 days of vacuum sealing drainage

No adverse effects, allergic reaction, or any type of side effect was observed in women who received VSD (none were observed in I&D patients either). A representative image of a patient who received VSD is shown in Fig. 3.

## Discussion

VSD is an treatment alternative that has been shown to promote the healing of complicated wounds, such as osteofascial compartment syndrome and dermatoplasty of large areas of cutaneous defects [6, 7]. However, the method has not been examined with respect to the treatment of acute suppurative mastitis. The results of this study showed that women with acute suppurative mastitis treated with VSD healed faster and experienced less pain at wound dressing changes than women treated with traditional I&D. This finding was also seen in women who were lactating. Our results are consistent with a prior literature review of the use of VSD for the treatment of complicated breast wounds such as those due to cancer and breast reconstructive surgery [11]. The review indicated that of 154 women with breast wounds treated with VSD, only 2 did not respond to the treatment and subsequently required flap coverage to treat the wound.

VSD is based on a number of simple and basic principles [12–15]. (1) The VSD material that covers the wound allows thorough wound drainage while not causing obstruction of the drainage tube. (2) The semipermeable membrane that covers the wound and VSD material prevents infection and promotes skin integrity. (3) Continuous negative pressure promotes local microcirculation and the growth of granulation tissue, and keeps the wound relatively dry.

The results of this study showed that VSD resulted in faster healing and less pain than conventional I&D. VSD also provides a number of other advantages compared to I&D. With VSD it is not necessary to change the wound dressing daily. In this study dressing were changed every 3 days in the VSD group, and wounds healed faster. Reducing the number of dressing changes markedly decreases the time needed from hospital personnel. Although not a variable in this study, VSD resulted in faster resolution of initial pain, redness, and swelling than I&D. As can be seen in Fig. 1, breast redness and swelling were almost completely resolved after only 3 days of VSD. In addition, in most patients there was obvious growth of granulation tissue after only 3 days of VSD (Fig. 1). Li et al. [6] reported that VSD enhanced blood circulation and stimulated tissue proliferation and repair when used for treating osteofascial compartment syndrome.

Our results did not show a difference in the length of hospital stay between patients treated with VSD and those treated with I&D. A possible explanation is that there are no uniform discharge criteria. Patients that live close to the hospital are likely to be discharged as soon as any evidence of infection has resolved and clear wound healing is evident, with follow-up care and dressing changes by a local healthcare provider. On the other hand, patients who live a great distance from the hospital, do not have access to a local care provider, and/or do not have a family support system are likely to be kept in the hospital longer than is medically necessary. In addition, medical insurance coverage may dictate the length of hospital stay, and patients without healthcare insurance will typically ask to be discharged as soon as possible.

We also did not find a difference in the length of antibiotic treatment between patients treated with VSD and those treated with I&D. This finding is not unexpected, and likely due to a number of different factors. All patients were treated with antibiotics, and the endpoint for discontinuing antibiotic treatment was the same for all patients (resolution of the breast mass, normal body temperature, normal WBC count). Both I&D and VSD have the effect of removing purulent material from an abscess, and the time required for this to occur appears to be the same with both methods. Yu et al. [16] reported that the length of antibiotic treatment was not different in patients with non-suppurative mastitis compared to those with suppurative mastitis treated with I&D. Second, there were no differences between the 2 groups with respect to baseline characteristics (e.g., age), type of abscess (e.g., plasma cell vs. not plasma cell), or initial disease severity. Lastly, suppurative mastitis is typically associated with a *Staphylococcus aureus* infection [8], and thus there was no difference in the antibiotics used or the treatment course between the groups.

An important finding of this study is that VSD was more effective in lactating women than I&D with respect to less pain and faster healing. This is important because acute mastitis is generally caused by incorrect breastfeeding, lack of breastfeeding experience, and poor drainage of breast milk, resulting in accumulation of breast milk and subsequent bacterial infection [17]. Breast milk is a natural culture medium for bacteria, and the secretion of milk can result in delayed wound healing. VSD addresses this issue by the removal of excess breast milk (a natural culture medium), removal of bacterial toxins, and the promotion of circulation and new tissue growth.

There are limitations of this study that should be considered. This was a retrospective analysis, and many patients were asked to recall healing time during a telephone interview, which may be subject to recall bias. Patients were not randomly assigned to the different treatments, they chose which treatment they preferred after discussion with their physician. Lastly, the number of patients was relatively small. While the results of this study are very promising, a prospective, random study with a larger number of patients is needed to confirm the results.

## Conclusions

This study showed that VSD is useful for the treatment of breast abscesses, and compared to traditional I&D is associated with less pain at wound dressing changes and faster healing. Importantly, the benefits of VSD were seen in lactating, as well as, non-lactating women. Due to the limitations of our experiment and sample size, prospective studies with large cohorts of patients are needed to confirm our findings.

## Data Availability

The datasets used and analysed during the current study are available from the corresponding author on reasonable request.

## Abbreviations

VSD: Vacuum sealing drainage
I&D: Incision and drainage
NRS: Numerical rating scale
WBC: White blood cell
IQR: Interquartile range

## References

[1] Dixon JM (2013). Breast infection. BMJ.

[2] Benson EA (1989). Management of breast abscesses. World J Surg.

[3] Leclercq A, Labeille B, Perrot JL, Vercherin P, Cambazard F (2016). Skin graft secured by VAC (vacuum-assisted closure) therapy in chronic leg ulcers: a controlled randomized study. Ann Dermatol Venereol.

[4] Ellis G (2016). How to apply vacuum-assisted closure therapy. Nurs Stand.

[5] Zhao XF, Li CY, Jin GQ, Ming XF, Wang GJ (2014). Vacuum sealing drainage combined with free skin graft in repairing cutaneous deficiency of traumatic shank amputation stump. Zhongguo Gu Shang.

[6] Li W, Ji L, Tao W (2015). Effect of vacuum sealing drainage in osteofascial compartment syndrome. Int J Clin Exp Med.

[7] Li Z, Wu W, Liu S, Hao Y (2017). Effect of vacuum sealing drainage in dermatoplasty of large area of cutaneous defects. Int J Surg.

[8] Angelopoulou A, Field D, Ryan CA, Stanton C, Hill C, Ross RP (2018). The microbiology and treatment of human mastitis. Med Microbiol Immunol.

[9] Wei J, Zhang J, Fu D (2016). Negative suction drain through a mini periareolar incision for the treatment of lactational breast abscess shortens hospital stay and increases breastfeeding rates. Breastfeed Med.

[10] Chen Y, Liu L (2016). Clinical analysis of 54 cases of large area soft tissue avulsion in the lower limb. Chin J Traumatol.

[11] Kostaras EK, Tansarli GS, Falagas ME (2014). Use of negative-pressure wound therapy in breast tissues: evaluation of the literature. Surg Infect (Larchmt).

[12] Qu J, Yan R, Wang L, Wu J, Cao L, Zhao G (2013). Free dermatoplasty combined with vacuum sealing drainage for the treatment of large-area soft tissue defects accompanied by bone exposure in the lower leg. Exp Ther Med.

[13] Yang YH, Jeng SF, Hsieh CH, Feng GM, Chen CC (2013). Vacuum-assisted closure for complicated wounds in head and neck region after reconstruction. J Plast Reconstr Aesthet Surg.

[14] Liu L, Tan G, Luan F, Tang X, Kang P, Tu C (2012). The use of external fixation combined with vacuum sealing drainage to treat open comminuted fractures of tibia in the Wenchuan earthquake. Int Orthop.

[15] Li RG, Yu B, Wang G, Chen B, Qin CH, Guo G (2012). Sequential therapy of vacuum sealing drainage and free-flap transplantation for children with extensive soft-tissue defects below the knee in the extremities. Injury.

[16] Yu Z, Sun S, Zhang Y (2018). High-risk factors for suppurative mastitis in lactating women. Med Sci Monit.

[17] Fetherston C (2001). Mastitis in lactating women: physiology or pathology?. Breastfeed Rev.

